# Depressive symptoms during rehabilitation period predict poor outcome of lumbar spinal stenosis surgery: A two-year perspective

**DOI:** 10.1186/1471-2474-11-152

**Published:** 2010-07-06

**Authors:** Sanna Sinikallio, Soili M Lehto, Timo Aalto, Olavi Airaksinen, Heikki Kröger, Heimo Viinamäki

**Affiliations:** 1 Department of Rehabilitation, Kuopio University Hospital, Kuopio, Finland; 2 Department of Psychiatry, University of Eastern Finland and Kuopio University Hospital, Kuopio, Finland; 3 Kyyhkylä Rehabilitation Center, Mikkeli, Finland; 4 Department of Orthopaedics and Traumatology, Kuopio University Hospital and Bone and Cartilage Research Unit, University of Eastern Finland, Kuopio, Finland

## Abstract

**Background:**

Previous research has shown an association between preoperative depressive symptoms and a poorer surgery outcome in lumbar spinal stenosis (LSS). It is not known whether depressive symptoms throughout the recovery period are relevant to the outcome of surgery in LSS. In this prospective clinical study the predictive value of preoperative and postoperative depressive symptoms with respect to the surgery outcome is reported.

**Methods:**

96 patients (mean age 62 years) with symptomatic lumbar spinal stenosis underwent decompressive surgery. They completed the same set of questionnaires preoperatively and 3 months, 6 months, 1 year and 2 years postoperatively. Depressive symptoms were assessed with the 21-item Beck Depression Inventory. Physical functioning and pain were assessed with the Oswestry Disability Index, the Stucki Questionnaire, self-reported walking ability and VAS rating. Logistic regression analyses were used to examine the predictive value of preoperative and postoperative depressive symptoms regarding the surgery outcome. A "good" outcome was defined in two ways: first, by gaining a 30% improvement in relation to the preoperative disability and pain, and second, by having a score at or below the median value for disability and pain on 2-year follow-up.

**Results:**

Having elevated depressive symptoms particularly on 3-month follow-up was predictive of a poorer surgery outcome regarding pain and disability: when the outcome was defined as less than 30% improvement from the baseline, the OR's (with 95% confidence intervals) were 2.94 (1.06-8.12), <0.05 for Oswestry and 3.33 (1.13-9.79), <0.05 for VAS. In median split approach the OR was 4.11 (1.27-13.32), <0.05 for Oswestry. Predictive associations also emerged between having depressive symptoms on 6-month and 1-year follow-ups and a poorer outcome regarding disability. The predictive value of elevated depressive symptoms particularly with respect to 2-yeard disability was evident whether the outcome was defined as a 30% improvement compared to the preoperative status or as belonging to the better scoring half of the study population on 2-year follow-up.

**Conclusions:**

Preoperative and postoperative depressive symptoms may indicate those patients at greater risk of a poorer postoperative functional ability. For these patients, further clinical evaluation should be carried out, especially during postoperative stages.

## Background

Lumbar spinal stenosis (LSS) is a disabling and painful disease that most typically affects middle-aged and older adults [1,2]. With the aging of the population, this condition is becoming increasingly common, and the rates of surgical treatment are increasing internationally [3,4]. The effectiveness of surgery for LSS has been found reasonably good regarding the most severe cases [5-7], but the success rates vary considerably [8,9].

The factors that influence the outcome remain controversial. Several research groups have tried to identify predictors of the surgical outcome of LSS [10,11]. The most often studied set of predictors includes biological, sociodemographic, work-related, psychosocial and general medical factors [8,12]. Generally, previous studies have suggested that the surgery outcome is related to preoperative factors [9]. However, preoperative prediction of the surgical outcome using self-rated measures of pain and disability or measures relating to the pathophysiology of the disease has been found difficult [9,11,13].

Interestingly, recent research has shown that depressive symptoms have direct biological effects on wound healing and pain among surgical patients through neuroendocrine-immune alterations [14-16]. It has also been found that there are high levels of interleukin-6, an inflammatory cytokine typical for depression [17], in facet joint tissue in degenerative lumbar spinal disorders (LSS and lumbar disc herniation) [18].

Previous research has suggested an association between depressive symptoms and a poorer surgery outcome [10,13,19], although studies of LSS and depression are scarce. In our earlier work we observed that on 3-month follow-up, preoperative depression among LSS patients was fairly persistent and that persistent depression was significantly associated with disability and pain [20]. We have also found that depressive symptoms in both preoperative and early recovery phases were strong predictors of the surgery outcome 1 year postoperatively [21].

As previous research has mainly focused on the development of pre-screening outcome tools [12], the temporal aspect of potential predictors has remained largely unknown. Although there is some evidence of the detrimental effect of preoperative and postoperative depressive symptoms on the LSS surgery outcome, it is not known whether depression plays a different role as an outcome predictor in different phases of the recovery process. Therefore, we performed a study to examine the predictive value of preoperative and early postoperative depressive symptoms with respect to the surgery outcome, using two different ways of defining the good outcome: first, by gaining a 30% improvement compared to the preoperative status [22], and, second, by having a score at or below the median value for disability and pain on 2-year follow-up. From a clinical point of view, recognizing depressive symptoms as a risk factor for poor outcomes may indeed yield timely clinical assessment and intervention practices. The principal hypothesis was that both pre-and postoperative depressive symptoms are associated with a poorer surgery outcome.

## Methods

The selection of the sample has been described in more detail by Sinikallio et al. [20,23]. Briefly, selection for surgery was performed by the orthopaedist or neurosurgeon at Kuopio University Hospital, Finland, between October 2001 and October 2004. The inclusion criteria were: 1) the presence of severe back, buttock, and/or lower extremity pain, with radiographic evidence (computed tomography, magnetic resonance imaging, myelography) of compression of the cauda equina or exiting nerve roots by degenerative changes (ligamentum flavum, facet joints, osteophytes and/or disc material); and 2) the surgeon's clinical evaluation that the patient had degenerative lumbar spinal stenosis requiring operative treatment. In addition, all patients had a history of ineffective responses to conservative treatment.

The exclusion criteria were: emergency or urgent spinal surgery precluding recruitment and protocol investigations; cognitive impairment prohibiting completion of the questionnaires or other failures in co-operation; and the presence of metallic particles in the body preventing the MRI investigation. A previous spine operation or co-existing disc herniation were not exclusion criteria, but the main diagnosis of the study patients had to be LSS. The surgeons sent the information on patients eligible for operation to the Department of Physical and Rehabilitation Medicine, which organized the study.

The patients received an account of the study during their outpatient visit to the Department of Physical and Rehabilitation Medicine and provided informed consent. The study design was approved by the Ethics Committee of the University of Kuopio and Kuopio University Hospital.

### Questionnaires

Preoperatively, questions concerning socio-demographic background, lifestyle and health were included in the study questionnaire. Data collection took place with the same set of questionnaires before surgery and 3 months, 6 months, 1 year and 2 years postoperatively. The questionnaire included the following items. **1) **Self-reported walking capacity was recorded. The patients gave an estimate of their walking capacity in metres by answering the question: How long a distance can you walk continuously without pausing, on even ground? **2) **Overall back and leg pain intensity was assessed with the visual analogue scale (VAS: range 0-100 mm) [24]. The overall back and leg pain of study subjects was enquired and recorded (with 0-100 mm VAS) at the preoperative and follow-up visits to the study physician. **3) **Subjective disability was measured by the validated Finnish version of the Oswestry Disability Index (0-100%), where 0% represents no disability and 100% extreme debilitating disability [25-27]. **4) **The questionnaire devised by Stucki [28] assessed LSS-related symptom severity, physical disability and postoperative satisfaction, with higher scores indicating more LSS-related problems and dissatisfaction. The questionnaire was translated into Finnish by one of the authors (TA) and a native English speaker checked the translation. There are currently no published validation studies using the Finnish version of the Stucki questionnaire. Two of the three Stucki scales were used in this study: 1) The symptom severity subscale is a 7-question scale on LSS-related symptom severity where all items except for one have Likert response scales with 5 categories scored 1-5 (none; mild; moderate; severe; very severe). The score was calculated as an unweighted mean of all answered items; 2) The physical disability subscale is a scale of LSS-related physical disability, where all items except for one have Likert response scales with 4 categories (no, could not perform; yes, but always with pain; yes, but sometimes with pain; yes, comfortably). The score was calculated as an unweighted mean of all answered items. The possible range of scores was 1 to 4. The third subscale on postoperative satisfaction was not included in this study.

**5) **Depression was assessed with the Finnish version of the 21-item Beck Depression Inventory (BDI), with scores ranging from 0 to 63 [29-32]. The cut-off point for depression was set at 9/10, 0 to 9 indicating normal mood and 10 or more indicating elevated depressive symptoms [29-31].

### Statistical analyses

All statistical analyses were performed using SPSS/PC (version 16.0., SPSS, Chicago IL, USA). The Student's t-test was used to compare the difference in mean scores between the groups in drop-out analysis. Logistic regression analyses were used to investigate the predictors for the surgical outcome, including preoperative and postoperative depressive symptoms. The rationale for predictor selection was based on two *a priori *defined points: 1) the well-known risk factors for depression were included, as well as 2) the clinically relevant factors regarding disability and pain. Regression analyses were performed using the data for the final 96 subjects who completed the two-year follow-up. A "good" outcome was defined in two ways: first, by gaining a 30% improvement compared to the preoperative disability and pain ("minimal important change")[22], and second, by having a score at or above the median value for disability and pain on 2-year follow-up ("median split").

First, univariate logistic binomial regression analysis was performed to investigate the unadjusted odds ratios of predictors at different follow-ups with respect to the surgery outcome, defined in the two different ways. Each of the predictors was entered into the model separately. We used four separate models to specifically examine the preoperative and recovery phase depression variables (model 1: preoperative predictors; model 2: 3-month predictors; model 3: 6-month predictors; model 4: 12-month predictors). The following factors were included as the basic covariates in the multivariate logistic regression analyses, regardless of the model: age (years), male (no/yes) and single (no/yes) as categorical variables. In addition, the Stucki symptom severity score, Stucki disability score and having elevated depressive symptoms (BDI ≥ 10: no/yes) were included in the models, according to the follow-up phase. The same set of predictors was used in all the logistic regression analyses.

Secondly, in the minimal important change approach, logistic regression analysis (with method enter; entering all variables at the same time) was used to examine the depression score at different follow-up points as a predictor with respect to a 30% decrease from the individual baseline disability (Oswestry score) and pain (VAS score). In practice, a "theoretical" 30% improvement was calculated as 30% decrease from the patient's baseline VAS score and Oswestry score. This calculation was done for each patient separately. Then each patient's actual 2-year score on VAS and Oswestry was compared to the "theoretical" outcome. In case the patient reached lesser than 30% decrease he was classified as having "poor" outcome regarding pain and disability.

Thirdly, in the median split approach, corresponding analyses with similar predictor models were performed.

## Results

### Study sample and drop-out analysis

The study subjects were 102 patients with both clinically and radiologically defined lumbar spinal stenosis (LSS) selected for surgical treatment. Beck Depression Inventory (BDI) data were missing at baseline for two of the study patients (n = 100) and at the 3-month follow-up for 3 of the 102 baseline patients (n = 99). At the final 2-year follow-up, four patients had died, one patient had dropped out of the study and one patient had missing BDI data (n = 96).

In drop-out analysis regarding the main demographic (age, gender) and clinical (number of somatic comorbidities, BDI score, Oswestry score) preoperative variables, there were no significant differences between the groups (the drop-outs, the dead, and the study group, data not shown).

The main diagnosis of all the study patients was LSS. Thirteen patients in our sample also had radiological disc herniation (DH) in addition to LSS. In the surgery, 19 patients were treated with spondylodesis with or without instrumentation. Preoperatively, the mean BDI score was 8.8 among patients treated with spondylodesis and 10.6 among patients without spondylodesis (p = ns.)

The mean age of the study patients preoperatively was 62 (SD 11.1) years. Altogether, 41% of the patients were male and 66% were married or living with a partner. Two study patients reported having used antidepressant medication at the preoperative stage, 3 patients at the 1-year postoperative stage and 7 patients at the 2-year postoperative stage. The clinical characteristics of the study patients in different follow-up phases are presented in Table 1.

**Table 1:** Preoperative, 3-month, 1-year and 2-year follow-up clinical characteristics of the LSS patients, n = 96.

	*PREOPERATIVE PHASE*	*3-MONTH FOLLOW-UP*	*6-MONTH FOLLOW-UP*	*1-YEAR FOLLOW-UP*	*2-YEAR FOLLOW-UP*
Stucki severity score (mean(SD)):	3.3 (0.6)	2.4 (0.7)	2.4 (0.8)	2.5 (0.8)	2.5 (0.7)
Stucki disability score (mean(SD)):	2.5 (0.5)	1.8 (0.5)	1.7 (0.6)	1.8 (0.6)	1.8 (0.7)
Oswestry score (mean(SD)):	44 (15)	26 (18)	24 (18)	27 (20)	26 (19)
VAS score, mm. (mean(SD))	32 (23)	18 (21)	26 (26)	19 (23)	12 (17)
walking capacity; meters (mean(SD)):	1464 (1818)	2440 (2263)	2880 (3062)	3063 (3651)	2728 (2963)
Mean BDI score among all the patients(mean(SD))	10.2 (6.0)	7.8 (5.7)	6.8 (6.5)	8.7 (7.3)	7.6 (5.9)
Proportion of patients having elevated depressive symptoms (%)(BDI score ≥ 10)	48	34	26	36	29

First, in univariate regression models, all the predictors were entered into the model separately (Table 2). In these models, having elevated depressive symptoms (on 3-month, 6-month and 1-year follow-up) predicted a less than 30% decrease in the disability score. Having elevated depressive symptoms (on 3-month and 6-month follow-up) predicted a less than 30% decrease in pain. Finally, a higher age, more severe LSS-related symptoms, more severe LSS-related disability and having elevated depressive symptoms (on 3-month, 6-month and 1-year follow-up) predicted greater 2-year disability.

**Table 2:** Logistic regression models showing unadjusted odds ratios (with 95% confidence intervals) of predictors at different follow-ups with respect to surgery outcome with each of the predictors entered into the model separately.

*Factor list*	*Osw decrease less than 30% from baseline: no/yes*	*VAS decrease less than 30% from baseline: no/yes*	*Osw ≥ median on 2-year follow-up* *(24): no/yes*	*VAS ≥ median on 2-year follow-up (0): no/yes*
**Model 1, preoperative:**				
Age (years)	1.01 (0.97-1.04)	1.00 (0.96-1.04)	1.04 (1.00-1.09)*	0.99 (0.95-1.02)
Gender (male: no/yes)	1.54 (0.67-3.58)	1.79 (0.74-4.34)	0.72 (0.32-1.63)	1.32 (0.58-3.01)
Marital status (single: no/yes)	1.45 (0.61-3.46)	1.31 (0.52-3.29)	1.71 (0.72-4.06)	1.53 (0.64-3.65)
Stucki symptom severity (continuous score)	1.02 (0.49-2.15)	0.82 (0.37-1.80)	2.46 (1.13-5.35)*	2.21 (1.01-4.84)*
Stucki disability (continuous score)	1.66 (0.67-4.12)	0.49 (0.19-1.26)	4.65 (1.70-12.71)**	2.53 (0.99-6.45) (p = 0.05)
**Depressive symptoms (BDI ≥ 10: no/yes)**	2.13 (0.90-5.00)	1.14 (0.47-2.78)	5.42 (2.23-13.16)***	1.45 (0.63-3.33)
^◆)^**Model 2, 3-month follow-up:**				
Depressive symptoms (BDI ≥ 10: no/yes)	4.29 (1.74-10.62)**	3.46 (1.36-8.81)**	4.88 (1.89-12.58)**	1.64 (0.68-3.90)
**Model 3**, **6-month follow-up:**				
Depressive symptoms (BDI ≥ 10: no/yes)	8.82 (3.01-25.86)***	2.81 (1.04-7.59)*	11.04 (2.99-40.74)***	3.43 (1.24-9.48)*
**Model 4, 1-year follow-up:**				
Depressive symptoms (BDI ≥ 10: no/yes)	4.84 (1.92-12.15)**	2.33 (0.93-5.88)	5.94 (2.27-15.55)***	1.51 (0.63-3.62)

^◆)^Only the significant odds ratios and those related to depressive symptoms are presented in models 2-4; *P < 0.05; **P < 0.01, ***P < 0.001

Secondly, in multivariate regression analyses (Table 3), when defining a "poor" outcome as less than 30% improvement from the baseline Oswestry and VAS score, the only significant associations emerged between the depression variables and the outcome: having elevated depressive symptoms (on 3-month, 6-month and 1-year follow-up) independently predicted less improvement in disability. In these analyses, having elevated depressive symptoms on 3-month-follow-up also independently predicted less improvement in pain.

**Table 3:** Logistic regression models showing adjusted odds ratios (with 95% confidence intervals) of predictors at different follow-ups with respect to a less than 30% decrease in disability and pain from baseline: a multivariate analysis with all the predictors entered into the model at the same time.

*Factor list*	*Osw decrease less than 30%: no/yes*	*VAS decrease less than 30%: no/yes*
**Model 1, preoperative:**		
Age (years)	1.00 (0.96-1.04) p = 0.96	1.00 (0.96-1.05) p = 0.85
Gender (male: no/yes)	1.46(0.58-3.66) p = 0.43	1.52 (0.57-4.05) p = 0.41
Marital status (single: no/yes)	1.40 (0.54-3.59) p = 0.49	1.25 (0.45-3.43) p = 0.67
Stucki symptom severity (continuous score)	0.77 (0.30-1.96) p = 0.58	1.36 (0.48-3.85) p = 0.56
Stucki disability (continuous score)	1.50 (0.45-4.96) p = 0.51	0.30 (0.08-1.14) p = 0.37
**Depressive symptoms (BDI ≥ 10: no/yes)**	1.86 (0.72-4.81) p = 0.20	1.60 (0.57-4.48) p = 0.37
^◆)^**Model 2**, **3-month follow-up:**		
Depressive symptoms (BDI ≥ 10: no/yes)	2.94 (1.06-8.12)* p = 0.04	3.33 (1.13-9.79)* p = 0.03
**Model 3**, **6-month follow-up:**		
Depressive symptoms (BDI ≥ 10: no/yes)	4.94 (1.35-18.09)* p = 0.02	2.83 (0.77-10.42) p = 0.12
**Model 4**, **1-year follow-up:**		
Depressive symptoms (BDI ≥ 10: no/yes)	2.91 (0.99-8.53) p = 0.05	2.05 (0.71-5.93) p = 0.19

^◆)^Only the significant odds ratios and those related to depressive symptoms are presented in models 2-4; *P < 0.05; **P < 0.01, ***P < 0.001

Thirdly, in multivariate regression models (Table 4) with respect to a poorer 2-year outcome in terms of disability and pain, the following associations were found: In logistic regression model 1 (preoperative phase), having elevated depressive symptoms predicted greater 2-year disability and pain. In logistic regression models 2 and 3 (3-month and 6-month phases), having elevated depressive symptoms predicted greater 2-year disability. In addition, more severe LSS-related symptoms on 3-month follow-up predicted greater 2-year disability and pain. Moreover, more severe LSS-related disability on 1-year follow-up predicted greater 2-year disability and pain. Finally, on 6-month follow-up, a higher age predicted greater disability on 2-year follow-up.

**Table 4:** Logistic regression models showing adjusted odds ratios (with 95% confidence intervals) of depression as a predictor at different follow-up points with respect to disability and pain on 2-year follow-up: a multivariate analysis with all the predictors entered into the model at the same time.^♠^

*Factor list*	*Osw ≥ median (24): no/yes*	*VAS ≥ median (0): no/yes*
**Model 1, preoperative:**		
Age (years)	1.04 (1.00-1.09) p = 0.08	0.98 (0.94-1.02) p = 0.38
Gender (male: no/yes)	0.73 (0.27-1.98) p = 0.54	1.96 (0.74-5.18) p = 0.18
Marital status (single: no/yes)	1.31 (0.46-3.74) p = 0.62	2.15 (0.79-5.85) p = 0.13
Stucki symptom severity (continuous score)	1.46 (0.51-4.22) p = 0.48	1.97 (0.74-5.30) p = 0.18
Stucki disability (continuous score)	1.94 (0.49-7.70) p = 0.35	2.96 (0.84-10.38) p = 0.09
**Depressed (BDI ≥ 10: no/yes)**	4.53 (1.65-12.44)** p = 0.003	2.74 (0.99-7.58) p = 0.05
^◆)^**Model 2**, **3-month follow-up:**		
Stucki symptom severity (continuous score)	4.06 (1.31-12.55)* p = 0.02	4.73 (1.57-14.25)** p = 0.01
Depressed (BDI ≥ 10: no/yes)	4.11 (1.27-13.32)* p = 0.02	1.36 (0.45-4.11) p = 0.59
**Model 3, 6-month follow-up:**		
Age (years)	1.07 (1.01-1.14)* p = 0.02	0.98 (0.94-1.02) p = 0.34
Depressed (BDI ≥ 10: no/yes)	6.94 (1.12-43.06)* p = 0.04	1.05 (0.27-4.03) p = 0.95
**Model 4, 1-year follow-up:**		
Stucki disability (continuous score)	11.73 (2.27-60.46)** p = 0.003	4.59 (1.32-15.93)* p = 0.02
Depressed (BDI ≥ 10: no/yes)	3.30 (0.87-12.46) p = 0.08	1.18 (0.38-3.63) p = 0.78

^♠^A poorer outcome refers to the subject having a score at or above the median value for disability and pain on 2-year follow-up (median split); ^◆)^Only the significant odds ratios and those related to depressive symptoms are presented in models 2-4; *P < 0.05; **P < 0.01, ***P < 0.001

## Discussion

The main finding of the present study was that in LSS patients undergoing decompression surgery, elevated depressive symptoms were a strong predictor of the surgery outcome in multivariate regression analyses. This was evident whether the outcome was defined according to the minimal important change from baseline or according to scoring over the median for pain and disability on 2-year follow-up. Importantly, these associations were particularly evident on 3-month and 6-month follow-up, but not so on 12-month follow-up.

In univariate regression analyses, when analyzing the predictive factors separately, the predictive value of 3-month and 6-month depressive symptoms regarding the outcome for disability and pain also became evident. Interestingly, in these analyses, elevated depressive symptoms on one-year follow-up also showed strong associations with greater and less improved disability as outcome. In univariate regression analyses it was also seen that out of the preoperative predictors, a higher age, more LSS-related symptoms and disability as well depressive symptoms were predictive of greater disability on 2-year follow-up.

To our best knowledge, this is the first study to examine the predictive role of elevated depressive symptoms assessed in LSS patients in several follow-up phases. From a clinical point of view, these results highlight the particular importance of the early recovery period; postoperative depressive symptoms may indicate those patients at greater risk of a poorer surgery outcome. In our study sample, only a fraction of the patients reported having used antidepressant medication during the follow-up period. As our study was a prospective observational design among a clinical sample of surgically treated LSS patients, psychiatric assessment or treatment was not included in the study protocol. However, when looking at the reported frequencies of antidepressant use, one would be safe to assume that depression among this patient population may well have remained largely undetected.

Based on the original and relatively low cut-off point suggested by Beck and Beamesderfer [30] for detecting depression, the proportion of the patients having elevated depressive symptoms declined during the follow-up. Some of the patients we classified as having elevated depressive symptoms might not have received a psychiatric diagnosis such as major depression or dysthymia. While 48% of the LSS patients had depressive symptoms preoperatively, the proportion at the end of the follow-up decreased to 29%. This would seem to imply that at least for some of the patients, the recovery from depressive symptoms may have been associated with a decrease in pain and disability, which is to be expected after surgical treatment for a painful and disabling illness such as LSS. However, it is known that normal clinical depression does not adequately recover without appropriate treatment, although in the short-term, depressive symptomatology has been found to decrease by 10-15% without treatment [33].

The most important remaining question concerns the mechanism explaining our results. Regarding the association between postoperative depression and a poor surgery outcome, one explanation may be the altered motivational state and executive-type cognitive impairments associated with depression, including a low motivation for physical exercise and activity. However, factors other than those of a psychological nature may also affect our findings. Persistent elevation of low-grade inflammatory activity - a physiological state closely associated with the pathophysiology of depression - may have direct effects on the process of physical rehabilitation among LSS patients, including effects on wound-healing and pain [14,15]. Similar associations concerning inflammatory activity have also been found among patients undergoing spinal surgery [16].

Interestingly, there is growing evidence that antidepressive treatment may reduce the production of pro-inflammatory cytokines [34,35]. Although previous literature on back patients' depression and immune system function is very scarce, the studies among patients with major depression/depressive disorder suggest that it is the depressive symptoms, not the depression diagnosis, that is essential with respect to immune system function [34,35]. In addition, previous research has shown that although both depression and pain respond to adequate antidepressant therapy, many patients are primarily treated with pain-relieving drugs that have little intrinsic antidepressant effect [36].

Nonetheless, one must be cautious not to label patients as "lost cases" or exclude them from surgical treatment due to their depressive symptoms. Instead, the current findings point to the need for appropriate assessment and treatment practises regarding concurrent depressive symptoms of LSS patients. In particular, our results suggest that research investigating the treatment of depression in patients undergoing surgery for stenosis merits a high priority.

## Conclusions

Although definite causal conclusions cannot be drawn from our observational study setting, it may be concluded that elevated depressive symptoms are associated with an increased risk for a poor surgery outcome among LSS patients, whether the outcome was defined according to the minimal important change from baseline or according to scoring over the median in pain and disability on 2-year follow-up.

This association was evident particularly for patients with elevated depressive symptoms on 3-month and 6-month follow-up. Various pre-screening measures for an optimal outcome have been extensively studied in LSS research [10], but our results indicate that early postoperative measures are also very important, particularly with respect to elevated depressive symptoms. Therefore, treatment models that include the use of depression scales and appropriate treatment practises throughout the preoperative and early postoperative period are encouraged. In the future, randomized controlled studies on the treatment of depression in LSS patients are needed.

## Competing interests

The authors declare that they have no competing interests.

## Authors' contributions

SS performed the statistical analysis and drafted the manuscript.

TA, OA, SML, HK, and HV participated in acquisition of data and helped to draft the manuscript.

TA, OA, HK and HV also participated in the design of the study.

All authors read and approved the final manuscript.

## Pre-publication history

The pre-publication history for this paper can be accessed here:

http://www.biomedcentral.com/1471-2474/11/152/prepub
